# The Voluntary Notification of Consumption

**Published:** 1899-07-01

**Authors:** 


### The Voluntary Notification of Consumption.

We think it is time that a serious word of warning should be given to those enthusiastic members of our profession who are being led by their otherwise praiseworthy zeal for the suppression of tuberculosis to undertake the voluntary notification of consumption. We will admit at once that it is most desirable that medical officers of health should obtain early information as to the occurrence of this disease, not only with the object of ensuring cleanliness and disinfection, but also for the purpose of ascertaining in what localities tuberculosis mostly tends to breed, for phthisis is a disabling complaint which drives its victims far down in the social scale before releasing them by death, so that death-rates tell but little as to where tuberculous maladies originate. When, however, we ask what right the practitioner has to disclose to anyone, whether public official or not, facts regarding his patient which have come to his knowledge in the course of his professional attendance, we see how great are the difficulties in the way of any form of notification which is not imposed as a statutory duty, and does not take place under the sanction of, and, indeed, by compulsion of the law of the land.

The matter can properly be considered under two quite different heads—the ethical and the legal—both of which seem to lead to the same conclusion. Looking at it from the side of professional confidence, that seal of secrecy by which medical men feel themselves bound in regard to all matters which come to their knowledge in the course of their professional work, we can have no hesitation in saying that even such notification as is demanded by law comes as a severe strain to all those ethical traditions by which for long centuries members of the medical profession have felt themselves bound, and that to go voluntarily and tell abroad these private matters would be a distinct breach of professional ethics and a violation of the confidence which should be looked upon as the basis of all professional intercourse.. But if we choose to put on one side for the moment all questions of ethics, and regard the matter as a mere question of law, we find weighty reasons why medical men will do well to be very chary about falling in with any scheme of voluntary notification; and it is especially desirable that these should be carefully considered, because, when schemes of this sort are put forward, under high authority, as being of great public utility and importance, the unthinking public will regard with much impatience, and will accept with very ill grace, the refusal of medical men to fall in with them on grounds which are sure to be described as "merely professional." We would then warn our professional brethren, before they offer their adhesion to any voluntary scheme of notification, to carefully consider how far such communications with the medical officer of health as will be involved in notifying a case of tuberculosis will be regarded in the eye of the law as privileged, for without doubt, if not privileged, the medical man making them will be fully liable for all injury which may result to his patient from his voluntary and uncalled-for intervention.

It will perhaps be said that no injury is done to a man by being ticketed as a case of consumption, and perhaps if it were possible to teach the public what is necessary for preventing the spread of the disease without at the same time scaring them out of their wits with the idea that every person suffering from tuberculosis is a standing source of infection, this might be true. Unfortunately, so far as the crusade against tuberculosis has touched the great mass of the people at all, it has touched them in the direction of planting in their minds the most disastrous idea which could possibly have taken root there—namely, a dread of infection. It is all very well to say that a consumptive person is innocuous if proper precautions are taken; but to the man in the street, who takes what he calls "common sense views," the word infection is enough. It is the very thing which he most greatly dreads. He maintains that the best way of dealing with infection is to avoid it, and once he is made to believe that consumption is infectious he will decline to work in the same office with a consumptive clerk, in the same workshop with a consumptive workman, in the same factory with a consumptive operative. And who shall say that he is wrong? "Shall I," he says, "buy strawberries that have been coughed over by a consumptive salesman, or eat buns out of a paper bag that has been blown into by a consumptive confectioner's assistant? Certainly not, if consumption is infectious; and as to all you say about proper care, how am I to know that proper care is taken?" And so complaints are laid, and all these good people are discharged, and all with one accord seek damages from the officious doctor who, by his interference, led to their being ticketed as consumptives. Let the doctors remember the very old adage, "The least said the soonest mended," and if they are driven into a corner, and if sanitary officials ask urgently, in the name of the public weal, that they should notify cases of phthisis, let them ask the simple question, Will you indemnify me against all legal proceedings? There is not a sanitary authority in the country which would give such an indemnity.

